# Sixth Volume

**Published:** 1870-04

**Authors:** 


### The Bistoury.

ELMIRA, N. Y., APRIL, 1870.

The BISTOURY is published Quarterly, upon the 1st of April, July, October and January, at Fifty Cents a year in advance.

Club Rates:--$25, will be paid for every 100 subscribers.

### SIXTH VOLUME.

Here we are again, brighter and livlier than ever. We are fairly running over with valuable information for the people. We are much improved in typographical appearance, and pride ourself upon being the cheapest medical journal in the world.

In this number we send an envelope and a blank for every person who has taken the BISTOURY for the last year, and who has forgotten to pay for it. Remember we give you many times more than the worth of your money, therefore we are not at all sensitive about "dunning" you. So, when you see the white envelope in your number, open it—you will find a blank inside—if you have not paid for *last* year, inclose a dollar, fill up the blank, replace it in the envelope, seal, affix a stamp, place it in your post office, and a noble deed is done! You have then paid for your BISTOURY one year in advance, as you ought to! If you have paid for last year, inclose fifty cents for the present volume. If you don't want the BISTOURY longer, pay up for the *last* year and we'll stop it. Come, pay up, friends! We need your money to pay the expenses of this publication. If you *all* pay, we will have enough means to give you twice as handsome a journal next time. We are not publishing the BISTOURY to make money out of it, but to benefit the people! We have spent, and will continue to spend, every dollar we receive for subscriptions, in beautifying and enlarging the journal.

We hope our readers will show the BISTOURY to their friends and ask them to subscribe for it. We also call attention to our Club rates, on inside of cover. It will be seen that we offer large cash premiums to those who interest themselves in our behalf.

So bye! bye! and look out for a still better number in July.

